# Oxyphosphates

**Published:** 1900-05-15

**Authors:** W. V. B. Ames


### OXYPHOSPHATES.

BY W. V. B. AMES, D D S.

The cement-forming phenomena exhibited by bringing together certain chlorides, phosphates and sulphates, and certain bases, are usually referred to as peculiar, if not mysterious. Dental text-books generally, and works on general chemistry dismiss the phenomena without attempting an explanation of the peculiar hardening process which ensues under proper conditions.

I will presume to place myself and you in a position to consider these cements tangibly by assuming that the hardening or setting is simply the result of the formation of basic salts. The formation of basic salts is sufficiently familiar to us. In adding an excess of the oxide of any negative metal to an acid which is a solvent of that oxide there will be instead of complete solution the natural equivalent, a partial solution and then the formation of a basic salt, which will dissolve on addition of excess of acid but not so readily as would the oxide if added slowly and not crowded to the extent of causing the formation of a basic salt.

Thus from analogy we can assume that oxyphosphate, oxysulphate and oxychloride of zinc is nothing more nor less than a mass in which an excess of zinc oxide granules are held together by basic phosphate, sulphate, or chloride of zinc, more or less modified by other salts which happen to be in solution in the liquid portion of the cement.

Observations under the microscope of the differences of texture of cements, measurements for shrinkage and expansion and tests for strength have led me to believe that all or most of the differences found in these materials can be attributed to the differences in the basic salt giving the cement-making phenomena.

For example, the most common formula for oxyphosphate of zinc, the one almost universally copied by writers having occasion to use a formula for this material, is the one calling for the solution of glacial phosphoric acid in water, for the liquid. Glacial phosphoric acid is really a mixture of meta phosphoric acid and sodium phosphate, the latter being added in indefinite quantity to give the glassy form desirable for handling. When this is mixed with zinc oxide a double basic phosphate of zinc and sodium is formed which acts to cement together the excess of zinc oxide granules. This sodium zinc basic phosphate is a more porous, friable cementing substance than would be a straight basic phosphate of zinc, such as would be formed if pure orthophosphoric acid were used as the liquid. This, however, would not give the smooth plasticity and slow setting which is obtained by the addition of sodium phosphate. This salt is peculiar in imparting those qualities to cement, but unfortunately does not at the same time give density and integrity to the hardened mass.

A happy medium, however, of easy working and density of mass can be gotten by working into orthophosphoric acid the phosphates of some of the metals other than of the alkaline group, such as magnesium, zinc, aluminum, copper, silver, etc., which will impart desirable working qualities and a less porous texture than is found in the glacial phosphoric acid product. This difference in oxyphosphates which really divides them into two classes, the alkaline and non-alkaline, was first mentioned, I believe, by me, in a paper read before the Columbian Dental Congress in 1893. No special cognizance has been taken of this in the interim by others. Writings on the question have not been voluminous. It is a considerable satisfaction, however, to know that in some work being done by another for the International Congress at Paris, of this year, almost the identical opinions will be offered and the cements divided into the same two classes, one in which the liquid contains alkaline phosphates in solution and the other in which they are absent. Of the first, the brands are legion, of the second, the few can be counted on the fingers.

There has been considerable agitation for over a year, of the undesirable porosity in cements as started by Dr. E. K. Wedelstaedt. The few oxyphosphates showing little or no porosity can safely be said to be of the small minority or second class mentioned. It is safe to say that as a result of the present agitation, the manufacturers will be led to improve their products, since it has been demonstrated that impervious cements with good working qualities can be produced. With an impervious cement, which makes a mass of almost flinty texture such as some obtainable, I am satisfied that results can be obtained infinitely better than writers of a few years since would venture to promise.

When the liquid portion of the cement contains phosphates of *non*-alkaline metals there is apt to be considerable trouble from crystalization and care must be exercised to avoid this to as great an extent as possible. When the crystals can be easily liquified the objection only amounts to an annoyance. When the liquid only takes on a slight cast from the formation of minute crystals which will be in suspension or loosely settled at the bottom of the bottle from long standing, these can usually be disregarded if the bottle is shaken each time to evenly distribute. When crystals form which are not easily soluble by warming, and which adhere to the bottom or sides of the bottle, it is safe to say that the liquid is not up to the ideal intended by the manufacturer.

The use of this class of cements calls for greater caution in managing the ingredients, than with the stereotype alkaline phosphate cement. It is more essential that a non-corosive spatula be used, as free acid is more in evidence which will attack a steel spatula, forming phosphate of iron to the detriment of the mix, as iron is not one of the metals which can be worked into a cement liquid to advantage. A point which I hesitate to urge is the necessity of scrupulously avoiding the contamination of the liquid within the bottle, by inserting an unclean instrument of any sort. A glass rod or dropper, an orange wood point, a clean hard tooth-pick, or clean non corrosive spatula is better for removing the desired amount of liquid than dropping or pouring from the bottle, but be very careful to avoid contamination. The least particle of the cement powder gotten by carelessness into the liquid bottle will tend to cause crystalization.

The means of making the most durable cement filling, I will not presume to advance. I believe in mixing cements for fillings as stiff as they can be and yet have sufficient plasticity for thoroughly packing to the cavity, never inserting or attempting to pack a crumbly mass. I desire to have the cement attain a decided crispness before attempting to trim it, especially at the cervical margin, and then trim from center when practicable. I have had the utmost satisfaction in the use of tin foil as advocated by A. Booth Pearsall, for compressing and confining the cement, and for getting the occlusion where necessary, by having the patient bring the occluding teeth into contact upon a piece of tin while the cement is still plastic. For crown setting I can only say that the cement should be mixed as stiff as its working qualities will admit.

### DISCUSSION.

DR. F. A. CODDING: The teeth of many children between the ages of twelve and sixteen seem to be lacking in lime salts, or at least they are evidently not as hard and resistant as in later life. I have made much use of the cements in filling these teeth to tide them over this period when they are specially prone to succumb to caries, and later when they are more dense or at least harder, I fill them with more durable material.

The method of mixing a cement has much to do with the results obtained; almost every cement presents a peculiarity of its own in this respect. Some mix thin and set very hard and make excellent fillings, while others must be worked quite stiff or they do not seem to stand in the mouth. And yet the same treatment will produce entirely dissimilar results in different mouths. I mixed a cement filling to the consistence of thick cream and flowed it into a cavity and allowed it to set without any manipulation and take on a smooth surface and glassy appearance. After it had hardened I coated it with a thin covering of hot, adhesive wax before allowing it to become exposed to the saliva. After two years that filling is as perfect as the day it was made. In another case similarly treated, the cement was dissolved out in less than six months. Whether it was faulty manipulation or an unfavorable condition of the saliva or buccal secretions which makes the difference, I am unable to say. While cements are valuable expedients, they can not be depended on for definite periods; they require close and frequent inspections.

Dr. C. R. Rowley, of Chicago: I use Justi's cement, and depend upon it. Having learned to manipulate it I know about what I can do with it. The structure of the teeth does not vary, at least any variation would not influence the permanency of the cement. The solvent of the cement is extraneous, that is to say, it comes from the secretions of the mouth or from destructive agents appearing or generated in contact with the fillings In those localities where cement fillings habitually fail I prefer to use gutta-percha. I do not use cements in close proximity or in actual contact with exposed pulps, as I believe they devitalize the pulps.

The President: I find that pulps die more frequently when exposed to some cements than to others.

Dr. White: I do not care to place any cement in actual contact with an exposed pulp, or even very sensitive dentin. I always cover such conditions with a film of chloro-percha.

Dr. C. H. Funk: In making approximal fillings with cement I finish the cement fillings with strips as usual, and then work into the surface a little parafine. Apply the paraffine hot and rub it into the cement with the cloth or back side of the finishing strip. This will protect the filling for a few days and allow it a chance to completely harden by preventing access of the dissolving fluids of the mouth

Dr. Hoff: I am quite satisfied from my experience with cements of the oxyphosphate varieties that they not only threaten the vitality of pulps, but that they have a corroding action on the dentin as well. Within the past year I have been investigating more carefully the condition of the dentin under oxyphosphate fillings, and I have been surprised to find that in a majority of instances there is apparent softening of the tissues of the tooth under cement filling, and I have almost come to the conclusion that a protecting varnish of some kind is a necessary precedent to the application of oxyphosphate cements as a filling I formerly attributed this condition to lack of adaptability to the cavity walls or to porous cements, but I am satisfied that it more often is the result of the corrosive action of the cements. It is probably due to the phosphoric acid, which is the liquid of oxyphosphates. The manufacturers of oxyphosphate cements claim to use the preparations of phosphoric acid that do not produce corrosive action or coagulation. But as a matter of fact, all cements are more or less irritant, and they can not be mixed and adapted to carious cavities of the teeth without there being a considerable quantity of free phosphoric acid to act upon the exposed dentin or pulp; and I fear that either through carelessness on the part of the manufacturer, or change in the material after leaving the hands of the manufacturer, that the material is too often unfit to be placed in the teeth. It requires very nice manipulation to obtait a chemically pure preparation of phosphoric acid, and it also requires delicate handling to keep it so. At present it seems impracticable for us to get any light on this subject, as we can not ask our manufacturers to disclose their methods of manufacture; but there is a good opportunity for some good scientific man to render an invaluable assistance to the profession by investigating this whole subject.

